# Nanoparticle-Enhanced PVDF Flat-Sheet Membranes for Seawater Desalination in Direct Contact Membrane Distillation

**DOI:** 10.3390/membranes13030317

**Published:** 2023-03-09

**Authors:** Indira Chimanlal, Lebea N. Nthunya, Oranso T. Mahlangu, Bastian Kirkebæk, Aamer Ali, Cejna A. Quist-Jensen, Heidi Richards

**Affiliations:** 1 Molecular Sciences Institute, School of Chemistry, University of Witwatersrand, Private Bag X3, Johannesburg 2050, South Africa; indira17.chimanlal@gmail.com (I.C.); nthunyalebea@gmail.com (L.N.N.); 2 Center for Membrane Technology, Department of Chemistry and Bioscience, Aalborg University, Fredrik Bajers Vej 7H, 9220 Aalborg, Denmark; bski@bio.aau.dk (B.K.); aa@bio.aau.dk (A.A.); 3 Institute for Nanotechnology and Water Sustainability, College of Science, Engineering and Technology, University of South Africa, Florida Science Campus, Johannesburg 1709, South Africa; orathem@gmail.com

**Keywords:** carbon nanotubes, desalination, membrane distillation, nanoparticle modification, silica nanoparticles

## Abstract

In this study, hydrophobic functionalized carbon nanotubes (fCNTs) and silica nanoparticles (fSiO_2_NPs) were incorporated into polyvinylidene fluoride (PVDF) flat-sheet membranes to improve their performance in membrane distillation (MD). The performance of the as-synthesized membranes was evaluated against commercial reference polytetrafluoroethylene (PTFE) flat-sheet membranes. The water contact angle (WCA) and liquid entry pressure (LEP) of the PVDF membrane were compromised after incorporation of hydrophilic pore forming polyvinylpyrrolidone (PVP). These parameters were key in ensuring high salt rejections in MD processes. Upon incorporation of fCNTS and fSiO_2_NPs, WCA and LEP improved to 103.61° and 590 kPa, respectively. Moreover, the NP additives enhanced membrane surface roughness. Thus, an increase in membrane roughness improved WCA and resistance to membrane wetting. High salt rejection (>99%) and stable fluxes (39.77 kg m^−2^ h^−1^) were recorded throughout a 3 h process evaluation where 3.5 wt% NaCl solution was used as feed. These findings were recorded at feed temperature of 60 ℃. Evidently, this study substantiated the necessity of high feed temperatures towards high rates of water recovery.

## 1. Introduction

According to published literature, 2.2 billion people lack access to safe drinking water [1,2,3]. Moreover, a steady decline in the abundance of freshwater resources and deteriorating infrastructure exacerbate existing water demands, thus creating a worsening crisis [3]. Furthermore, developing countries particularly in the African continent experience intermittent water supply [4,5,6]. More often, consumers use potable water under restricted conditions where water rationing is implemented. Predominantly, access to safe drinking water is affected by poor financial reserves and inadequate infrastructure [5,7]. Rapid population growth, industrialization, and climate change threaten freshwater resources resulting in substantial water shortages.

In addition to water scarcity, mineral resources are depleting at an alarming rate [8,9]. Among other factors, the increasing use of renewable energy sources further intensifies this demand. This is exacerbated by a rising demand in mineral resources due to growing economic and industrial sectors [10]. For example, the production of solar panels requires elements such as cadmium, silver, or indium [11], while electric vehicle batteries require lithium, nickel, and cobalt [8]. These pressing issues motivate the need for improved and sustainable technology solutions. Among others, membrane distillation (MD) emerged as a promising technology to address these issues, albeit relatively novel [12,13]. Briefly, this technology utilizes a temperature difference between the two interfaces of the membrane, thus generating a vapour pressure gradient to drive this process [14,15]. Furthermore, a hydrophobic membrane is employed to exclusively retain mineralized feed water while extracting fresh water in the form of vapour [16,17]. Additionally, the MD is well suited for the treatment of industrial wastewater [18,19]. Importantly, this technology relies on its capability to treat harmful industrial wastewaters and waste recycling through the recovery of valuable constituents from feed streams [20,21,22]. Furthermore, MD is characterized by high permeate quality, simple operation configuration and process conditions, such as temperature and pressure. Interestingly, MD treats a variety of solutions independent of their concentration, thus producing fresh water from a myriad of feed streams [18,23,24,25]. Also, the MD is suited for resource recovery when coupled with crystallization, thus giving rise to membrane distillation crystallization (MDC). In this process, the feed solution is concentrated towards supersaturation, thus retrieving minerals from a waste solution. For instance, the MDC was evaluated for recovery of both minerals and water from shale gas where a low energy consumption of 28.2 kWh m^−3^ was used [26]. Other previous studies reported the recovery of minerals such as NaCl, CaCO_3_, BaCO_3_, and Na_2_SO_4_ [18,26,27]. Additionally, MD has demonstrated its compatibility in hybrid systems. Coupling of MD to various techniques has been reported to improve process performances. Scaling control was achieved through the integration of forward osmosis (FO) and MD. Another study reported a hybrid system composed of multi-effect evaporation and MD for solar energy desalination. The hybrid system presented improved process performance compared with a standalone evaporation system. Furthermore, the water production capacity was 159.84 m^3^ yr^−1^ [28]. However, these systems are costly. The MD advancement towards resource recovery (simultaneous recovery of fresh water and minerals) is imperative. However, existing MD membranes are prone to fouling and wetting leading to performance deterioration [29]. Accordingly, membrane cleaning protocols are employed. However, performance restoration is not guaranteed [30]. Membrane modification arose to address concerns surrounding fouling and wetting, thus exploring various additives. Moreover, modified membranes are characterized by improved structural integrity and selectivity among other physical properties [29]. Likewise, porous membranes are produced through modification processes. This is imperative because the mechanism for water vapour mass transport is primarily influenced by the membrane pores and is controlled by the Knudsen molecular diffusion [31].

In the current study, nanoparticle-enhanced PVDF membranes were fabricated and evaluated for the treatment of synthetic seawater. Previously, various additives were incorporated into hydrophobic polymeric membranes to enhance their resistance to fouling and wetting, thus improving the permeate flux and salt rejections [32,33,34]. Such modifications include the incorporation of silica nanoparticles (SiO_2_NPs) into a PVDF polymeric matrix to impart resistance towards scaling [35], surface fluorination to improve fouling resistance [36], and silver coating to improve flux [37]. Different from previously reported literature, the current study explored fluorination of the CNTs using a fluorinated silane reagent, with a subsequent embedment into PVDF membranes. Moreover, this study explored the synergistic effect of modified CNTs and SiO_2_NPs. Metal oxide additives such as SiO_2_NPs were used due to their advantages such as ease of functionalization and their ability to enhance the physical properties of membranes [29,38]. Similarly, the addition of CNTs offers durability and improved structural integrity [39,40]. The modified membranes were evaluated towards desalination of 3.5 wt% synthetic seawater in direct contact membrane distillation (DCMD).

## 2. Materials and Methods

### 2.1. Chemicals and Equipment

Tetraethyl orthosilicate (TEOS, reagent grade, 98.0%), ammonium hydroxide (NH_4_OH, ACS reagent, 28.0–30.0% NH_3_), absolute ethanol (ETOH, ACS reagent, 99.5%), toluene (anhydrous, 99.8%), sodium chloride (NaCl, ACS reagent, 99.0%), 1H,1H,2H,2H-perfluorooctyl triethoxysilane (POTS, MW = 610.38 g·mol^−1^, 97.0%), isopropanol (ACS reagent, 99.5%), nitric acid (HNO_3_, puriss p.a., 65.0%), polyvinylidene fluoride (PVDF, MW = 534,000 g·mol^−1^), and polyvinylpyrrolidone (PVP, MW = 360,000 g·mol^−1^, dimethylformamide (DMF, ACS reagent, 99.8%) N,N-dimetheylacetamide (DMAc, puriss p.a., 99.5%), were acquired from Sigma-Aldrich (Darmstad, Germany). Multiwalled carbon nanotubes (MWCNTs, outer diameter = 10 nm, 98.0%) were obtained from SabiNano (Pty) Ltd. (Johannesburg, South Africa). In addition, 2 commercial PTFE membranes (pore sizes of 0.45 µm and 0.20 µm) supported on non-woven polyester (LH0P) were procured from Pall Corporation (New York, NY, USA). The membranes were termed PTFE-20 (0.20 µm) and PTFE-45 (0.45 µm). Ultrapure water was obtained from our laboratory using Milli-Q-RO4 (Millipore, Bedford, MA, USA). Permeate conductivity was recorded using the Mettler Toledo conductivity probe (Columbus, OH, USA).

### 2.2. Synthesis and Functionalisation of Silica Nanoparticles (SiO_2_NPs)

The SiO_2_NPS were synthesised using a modified StÖber method [35]. Briefly, TEOS (10 mL) and ethanol (40 mL) were added to a mixture of de-ionized water (22.5 mL), NH_4_OH (10 mL), and ethanol (16.5 mL). This was followed by vigorous stirring until a milky-white turbid mixture was produced. The resultant mixture was stirred for 5 h at a slower speed followed by centrifugation in Hettich Zentrifugen Rotofix 32A (Tuttlingen, Germany) and washed with ethanol. Subsequently, it was dried at 50 ℃ for 24 h. The resultant SiO_2_NPS were dispersed in ethanol and bath sonicated (Eins Sci Profession ultrasonic cleaner, Johannesburg, South Africa) for 30 min to reduce particle aggregation. Furthermore, the nanoparticles (NPs) were filtered and dried. Prepared SiO_2_NPS were functionalized using 1H,1H,2H,2H-perfluorooctyl triethoxysilane (POTS). Briefly, SiO_2_NPS were dispersed in an appropriate volume of toluene followed by the slow addition of 40 wt% POTS using a syringe. The resultant material was stirred at room temperature for 5 hs. The functionalized nanoparticles were washed with ethanol to ensure the removal of any excess reagent followed by drying for 24 h. Functionalized SiO_2_NPS were termed fSiO_2_NPS.

### 2.3. Functionalisation of CNTs

Commercially acquired CNTs were functionalized to enhance their hydrophobic characteristics before dispersion into PVDF membranes. The functionalization was adopted from a modified method reported by Gao et al. (2020). The method was conducted in a two-step process namely, an acid treatment followed by fluorination using a fluorosilane reagent [41]. Unmodified CNTs were added to a 65% HNO_3_ and sonicated for 1 h followed by constant stirring for 24 h at ≈80 ℃. The resultant mixture was cooled, centrifuged, and washed with deionized water. The CNTs were dried and kept for further modification. Dried CNTs were added to 82 mL of toluene followed by sonication for 70 min to promote particle dispersion. Thereafter, a 0.5 wt% POTS solution containing toluene was added to the mixture containing CNT–toluene and stirred for 24 h. The functionalized CNTs were centrifuged, washed using toluene and dried in an oven at 60 ℃ for 24 h. The functionalized CNTs were termed fCNTs.

### 2.4. Membrane Preparation

An appropriate amount of PVDF was dissolved in a mixed solvent system of DMAc and DMF at the ratio of 2:3. The solution was stirred for 24 h followed by degassing. The resultant polymer solution was cast on a glass plate to an approximate height of 50 µm using an Elcometer 4340 casting knife film applicator (Manchester, U.K.). The cast solution was placed in a coagulation bath containing water (a non-solvent). To ensure complete phase separation, the membrane solution was kept in a coagulation bath for 2 days. A similar procedure was followed during the synthesis of M2, M3, and M4. The composition of each membrane is provided in Table 1. To prepare M3 and M4, the fCNTs and fSiO_2_NPs were sonicated in the solvent to ensure particle dispersion before addition to the polymer solution. Notably, the concentration of the fCNTs used in this study was chosen based on previously reported literature. According to Khalid et al. (2017), CNT loadings >0.4 wt% cause agglomeration, thus resulting in poor dispersion. This was justified by the strong van der Waal’s interactions between the carbon lattice of the CNT [42].

### 2.5. Characterisation of the NPs and Membranes

FTIR was used to understand the physicochemical characteristics of the synthesised NPs and membranes. Fourier-transform infrared spectroscopy (FTIR) analysis was conducted using a Bruker Tensor 27 FTIR (Billercia, MA, USA). Spectra were obtained at a wavelength range of 3900–650 cm^−1^ (Figure S1 and Figure S2). Transmission electron microscopy (TEM) was used to evaluate the morphological and particle size of pristine SiO_2_NPs and CNTs. All micrographs were recorded using FEI Tecnai T12 TEM at 120 kV (Hillsboro, OR, USA). Surface and cross-sectional morphologies of each membrane were obtained from the Zeiss EVO 60 scanning electron microscope GmbH (Oberkochen, Germany). Samples were coated with Au prior to analysis. Membrane roughness and topography were evaluated using atomic force microscopy (AFM). The AFM analysis was conducted using the WITec Alpha 300A TS-150 (WITec Wissenschaftliche Instrumente und Technologie GmbH, Ulm, Germany). Mechanical properties of the membranes were acquired from stress–strain plots obtained from the AG-Plus Universal tester (Shimadzu Europa GmbH, Duisburg, Germany). The size of the membrane specimen was 25 × 25 mm^2^ and the pulling speed was 0.5 mm min^−1^. The membrane water contact angle (WCA) was analysed through the sessile method using a drop shape analyser (Biolin Scientific Attention Theta Line, Stockholm, Sweden) to understand their hydrophobicity. The measurements were conducted at room temperature using deionised water. A dead-end filtration cell was used to measure the liquid entry pressure (LEP) of the membranes. A circular piece of the dry membrane (diameter = 5 cm) was placed in a dead-end filtration cell. Afterwards, the cell was filled with ultrapure water. To determine the minimum pressure required to eject the water, the pressure was gradually increased until the first water drop was produced. Membrane pore sizes were determined in POROLUX^TM^ 1000 using porefil as the wetting agent, and a membrane area of approximately 300 mm^2^. Membrane porosity was determined using a modified gravimetric method [39]. A dry membrane (*m_d_*) (1 cm^2^) was immersed in an appropriate volume of isopropanol for 24 h to ensure complete solvent absorption into the membrane pores. Following this, the mass of the wetted membrane was measured (*m_w_*). Each measurement was performed in triplicate. Equation (1) was used to determine the porosity of the as-synthesised membranes:

(1) $$\varepsilon =\frac{\left(\frac{m_{w}-m_{d}}{\rho_{solvent}}\right)}{\left(\frac{m_{w}-m_{d}}{\rho_{solvent}}\right)+\left(\frac{m_{d}}{\rho_{polymer}}\right)}\;\times \;100$$

where *m_w_* and *m_d_* are the masses of wet and dry membranes respectively, and $\rho_{solvent}$ and $\rho_{polymer}$ are the densities of isopropanol (0.786 g cm^−3^), PVDF polymer (1.78 g cm^−3^), and PTFE polymer (2.20 g cm^−3^), respectively.

### 2.6. Membrane Distillation

A laboratory-scale DCMD system was employed to evaluate seawater desalination using the as-synthesised membranes. Deionized water (dH_2_O) (Figure S3) and 3.5 wt% NaCl were used as feed solutions. These solutions were circulated at a feed temperature range of 40 ℃, 50 ℃, and 60 ℃. For the distillate, deionised water was circulated at a temperature of approximately 10.0 ℃. Across the flat-sheet membrane module (3.3 × 8.0 mm^2^), four temperature sensors were installed at the entrance and exit channels of both the feed and permeate. The feed and permeate were circulated in a co-current mode at the crossflow velocity of 601.0 mL·min^−1^. Although counter-current solution configurations are typically employed, the small membrane area used in this study meant that a co-current configuration would have negligible effects on the system performance. The weight increment and conductivity of the permeate were measured continuously. To ensure continuous dissolution of the NaCl feed solution, the solution was stirred continuously. After each experiment, the membrane was rinsed with deionized water to wash away any precipitated salts from the surface. Permeate flux (*J*) was calculated using Equation (2), where Δm is the permeate mass difference (kg), Δt is the time difference (hr), and A is the membrane area (m^2^).

(2) $$J=\frac{\Delta m}{\Delta t*A}$$

The temperature gradient in DCMD was estimated using the log mean temperature obtained from Equation (3), where ΔT_1_ and ΔT_2_ are defined by Equations (4) and (5), respectively.

(3) $$\Delta T_{\ln}=\frac{\Delta T_{1}-\Delta T_{2}}{\ln\left(\frac{\Delta T_{1}}{\Delta T_{2}}\right)}$$

(4) $$\Delta T_{1}=T_{\mathrm{retentate},\mathrm{in}}-T_{\mathrm{permeate},\mathrm{in}}$$

(5) $$\Delta T_{2}=T_{\mathrm{retentate},\mathrm{out}}-T_{\mathrm{permeate},\mathrm{out}}$$

## 3. Results and Discussion

### 3.1. TEM Analysis of the SiO_2_NPs and CNTs

**Figure 1:**
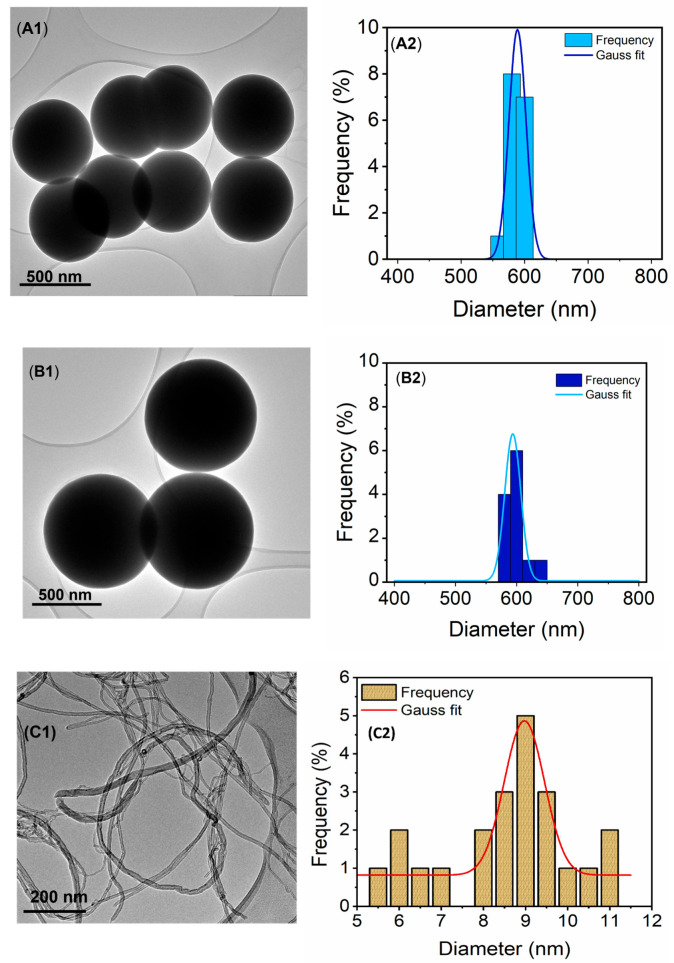

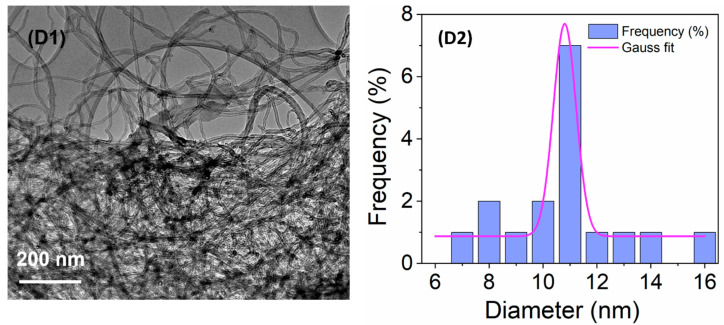
TEM micrographs and corresponding particle size distribution (**A1**,**A2**) SiO_2_NPs, (**B1**,**B2**) fSiO_2_NPs, (**C1**,**C2**) CNTs, and (**D1**,**D2**) fCNTs.

The TEM micrographs (Figure 1) were used to evaluate morphology, particle size, and distribution of the SiO_2_NPs (A1-A2), fSiO_2_NPs (B1-B2), CNTs (C1-C2), and fCNTs (D1-D2). The SiO_2_NPs and fSiO_2_NPs were homogenous and spherical with minimal structural defects (Figure 1A1,B1). The particle size of the SiO_2_NPs and fSiO_2_NPs were 588 ± 7.21 × 10^−3^ nm and 593 ± 7.49 × 10^−1^ nm, respectively (Figure 1A2,B2). Notably, a slight increase in the particle size was realized after silane modification of the SiO_2_NPs. This increase was attributed to the coating effect of the silane reagent (i.e., POTS).

The CNTs were elongated, homogenous, and cylindrical tubes with minimal irregularities (Figure 1C1,D1). Their sizes before and after modification were 8.97 ± 0.0969 nm and 10.8 ± 0.296 nm, respectively (Figure 1C2,D2). Similarly, an increase in the particle size was realized following modification. The increase confirmed silane growth on the surface of the CNTs. The modification process was further verified from the FTIR analysis (SI1).

### 3.2. Membrane Porosity, Pore Size, WCA, and LEP

The porosity measurements of M1, M2, M3, and M4 were 81.68%, 73.00%, 64.34%, and 63.61%, respectively (Table 2). Notably, M1 presented the highest porosity with a decreasing porosity upon incorporation of the additives (M2–M4). The decreasing porosity following modification was substantiated by the reported literature [43]. Furthermore, a decline in the porosity of M3 and M4 was largely due to membrane pore blockage caused by additive cavity filling. The porosity values of commercial PTFE-45 and PTFE-20 were 51.03% and 54.13%, respectively. These porosity values were lower than all the synthesised membranes. The discrepancy was associated with the difference in fabrication procedures and polymer types [44]. The pore sizes of M1 and M2 were 0.27 µm and 0.21 µm, respectively (Table 2). Upon incorporation of fCNTs (M3), the pore size decreased to 0.19 µm. A similar effect was realised for M4 (0.22 µm). A slight increase in pore size from M3 to M4 was caused by the incorporation of fSiO_2_NPs [39,45]. According to Fernandes et al. (2021), hydrophobic SiO_2_NPs increase solution viscosity as a result of a sudden change in polymer entanglement [46]. The polymer entanglement increases in the presence of water during phase inversion caused by the low affinity between the hydrophobic NPs and water. As per ternary diagrams, Alibakhshi et al. (2019) reported an increase in membrane pore size due to the reduced affinity of the polymer to the non-solvent during phase inversion [47]. The presence of hydrophobic NPs enables the steric hindrance of solvent and non-solvent exchange, leading to the formation of abundant and large pore sizes [46]. These processes justify the slight increase in membrane pore size from M3 to M4.

The WCA M1 was 116.52°, which decreased to 84.69° upon the addition of PVP in the casting solution (Table 2). The decrease in membrane hydrophobicity was associated with the hydrophilic nature of the water-soluble pore former (PVP). However, upon the addition of fSiO_2_NPs and fCNTs, the WCA of M3 and M4 increased to 101.60° and 103.81°, respectively, implying an improvement in their hydrophobicity. Similar results were reported by Silva et al. [39] upon incoporating multi-walled CNTs (MWCNTS) into a PVDF polymeric matrix. Though WCA increased upon the addition of fCNTs, membrane hydrophobicity remained relatively lower than prestine PVDF membrane, largely due to minimal fluourination of the CNTs. Compared with M3, the WCA of M4 improved due to the addition of fSiO_2_NPs. The WCA of PTFE-20 and PTFE-45 were 97.35° and 101.57° respectively, thus comparable to modified M3 and M4. These WCA values suggest the membrane’s suitability for use in MD applications. 

**Figure 2:**
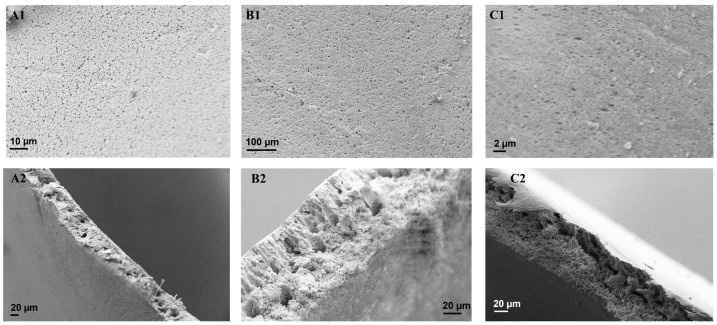

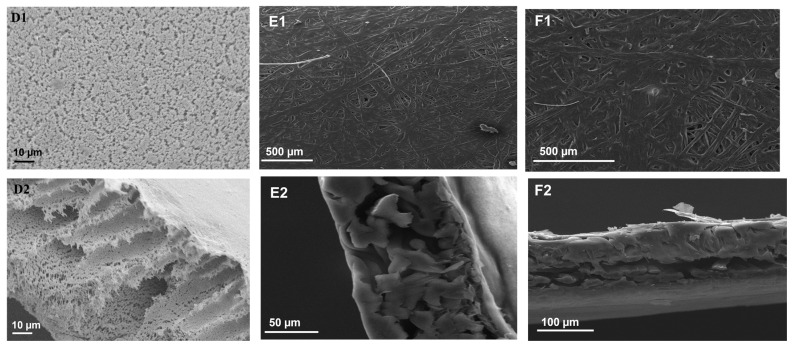
Surface and cross-sectional SEM micrographs of (**A1**,**A2**) M1, (**B1**,**B2**) M2, (**C1**,**C2**) M3, and (**D1**,**D2**) M4, (**E1**,**E2**) PTFE-20, and (**F1**,**F2**) PTFE-45.

Liquid entry pressure (LEP) measurements were carried out to understand the possible wettability of the membranes by the process liquids. The MD operating pressure should not exceed the LEP of the membranes to ensure their wetting resistance. The LEP of a membrane is governed by various factors including membrane pore size and hydrophobicity [48,49]. The LEP of M1 was 393 ± 4.00 kPa (Table 2). Upon the addition of the pore former, the LEP of M2 decreased to 273 ± 37.7 kPa. The drop in LEP was caused by the decline in mass transfer resistance. This was explained by its low WCA, therefore, its hydrophilic properties and larger pore size established minimal resistance to mass transfer through the membrane. However, the incorporation of the fCNTs and fSiO_2_NPs increased the LEP of M3 and M4 to 500 ± 97.9 kPa and 590 ± 90.0 kPa, respectively. The increased LEP of M3 and M4 was associated with an increase in mass transfer resistance caused by a decreased membrane pore size and improved hydrophobicity indicated by their WCAs. The reduced pore size and LEP was supported by previously reported studies [43,45]. The LEP of PTFE-20 and PTFE-45 were 603 ± 20.5 kPa and 200 ± 90.0 kPa, respectively. PTFE-45 was characterized by a larger pore size (i.e., 0.45 µm) compared with PTFE-20 (0.20 µm), thus enabling low mass transfer resistance. Moreover, membrane pore geometry is an important factor, as pore irregularities in axial and radial directions result in deviations from perfectly cylindrical pores, thus affecting the mass transfer [50]. Differences in LEP were correlated to SEM micrographs of these membranes (Figure 2C1,F1). Irregularly shaped pores were recorded with different circular, elongated, and cylindrical structures.

### 3.3. SEM Analysis of the Membranes

The obtained SEM micrographs were used to evaluate the surface and cross-sectional morphology of the as-synthesised PVDF membranes (Figure 2). A surface view of M1 (Figure 2A1) presented a densely porous sponge-like surface. Based on the cross-sectional micrograph (Figure 2A2), the membrane was characterized by smaller and round pores. Although M2 was densely porous, this membrane possessed a globule-like surface (Figure 2B1). Based on cross-sectional analysis (Figure 2B2), M2 was characterized by elongated, finger-like pore structures. This is a consequence of the addition of PVP into the membrane matrix [39]. Due to its high water solubility, PVP promoted the formation of elongated pores during phase separation [51]. Nonetheless, SEM does not indicate an increase in membrane porosity from M1 to M2, thus supporting the previously reported information (Table 2). For the modified membrane, M3 showed a densely porous structure with varying pore sizes (Figure 2C1,C2). The internal structure contained a complex mixture of small, round, spongy pores in addition to elongated pores (Figure 2C2). Similar findings of sponge-like pores were reported by Silva et al. [39]. The pore elongation was linked to the addition of fCNTs. Lastly, M4 (Figure 2D1) revealed a densely porous surface with an irregular pattern, caused by the addition of fSiO_2_NPS in the casting solution. Analysis of the corresponding cross-sectional view demonstrated a combination of elongated pores and small, dense pores induced by the addition of PVP, fCNTs, and fSiO_2_NPS, respectively. Notably, the internal structure of M4 contained macrovoids induced by membrane additives (Figure 2D2) [52]. PTFE-20 and PTFE-45 were characterized by rod-like structures with irregularly shaped pores (Figure 2E1,F1). Based on their cross-sectional views, these membranes were dense with an irregular porous structure.

### 3.4. AFM Analysis of the Membranes

**Figure 3:**
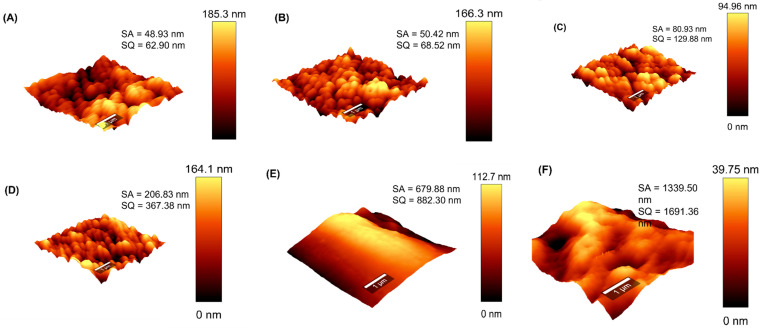
Topographical micrographs displaying surface roughness of (**A**) M1, (**B**) M2, (**C**) M3, (**D**) M4, (**E**) PTFE-20, and (**F**) PTFE-45.

Owing to its effect on process performance, the membrane surface roughness was evaluated and presented in Figure 3. The root-mean-square roughness (R_q_) for M1, M2, M3, M4, PTFE-20, and PTFE-45 were 62.90 nm, 68.52 nm, 129.88 nm, 367.38 nm, 882.30 nm, and 1691.36 nm, respectively. Modified membranes (M2-M4) displayed a greater density of dark and light ridges indicating the deepest and highest regions on the membrane surface (Figure 3B–D). Technically, these membranes presented superior surface roughness compared with M1 (Figure 3A) [53]. The R_q_ values increased upon the incorporation of the pore former and additives (PVP, fCNTs, and fSiO_2_NPS), suggesting increased surface roughness. The surface roughness of the commercial membranes PTFE-20 (882.3 nm) and PTFE-45 (1691.36 nm) exceeded that of the as-synthetic membranes. Based on the rough surface acting as air pockets, the performance of these commercial membranes may supersede that of the as-synthetic membranes in MD processes. Comparably, increased membrane surface roughness caused by the incorporation of NPs was reported in various studies [54,55].

### 3.5. Mechanical Properties of the Membranes

**Figure 4:**
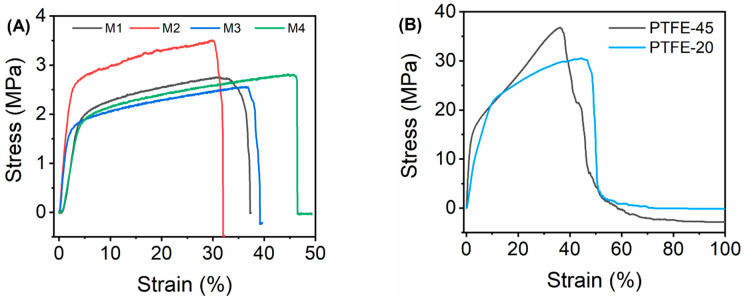
Stress–strain plots of (**A**) the as-synthesised membranes and (**B**) the commercial membranes.

The mechanical properties of the as-synthesised were determined using Young’s modulus estimated from the stress–strain plots (Figure 4A,B). Mechanically strong membranes are required to sustain MD operating conditions [56]. The tensile strength of the membranes was reported on the basis of Young’s modulus (Table 3). The Young’s modulus of M1 (0.45 MPa) was lower compared to M2 (1.06 MPa). This was attributed to the porous microstructure of M1, thus weakening its mechanical properties [57]. Moreover, membrane pores act as stress absorbers thus increasing the tensile strength of M2. Similar findings were reported by Pramono et al. [56]. There were no significant differences in the mechanical strengths of M2 (1.06 MPa) and M3 (1.07 MPa). However, the addition of fCNTs presented a slight increase in the mechanical strength of the membrane. The carbon atoms present in the single graphene of fCNTs are characterized by strong chemical bonds. These bonds increase the elasticity of the fCNTs, ensuring full restoration of the particle size upon release of the external force [58]. When incorporated into the polymer matrix, fCNTs increased membrane strength. However, the tensile strength of M4 decreased to 0.39 MPa upon the incorporation of fSiO_2_NPs. The decrease was a consequence of macrovoids causing structural defects of the membrane (Figure 2D2) [52,59,60]. Furthermore, nanoparticle aggregation cause skewed mechanical integrity of the modified membrane [57]. Lastly, the mechanical properties of the commercial membranes, PTFE-20 (2.90 MPa) and PTFE-45 (6.96 MPa), were higher compared to synthetic membranes. These differences were associated with the mechanical integrity of the different polymers.

### 3.6. Flux and Salt Rejection Evaluation in DCMD Using Synthetic Salt Water

**Figure 5:**
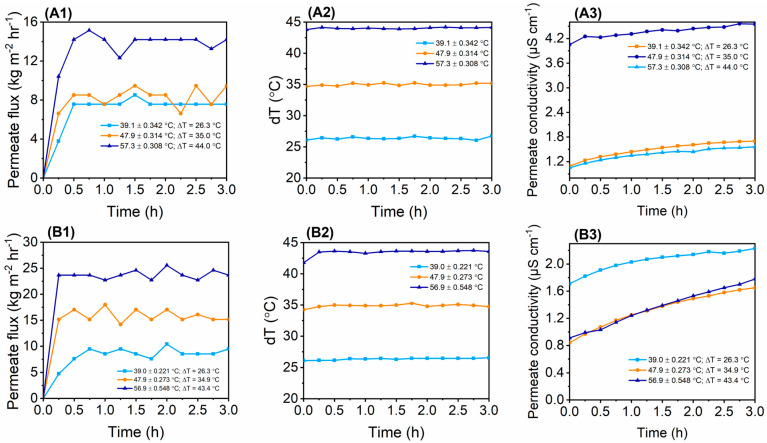

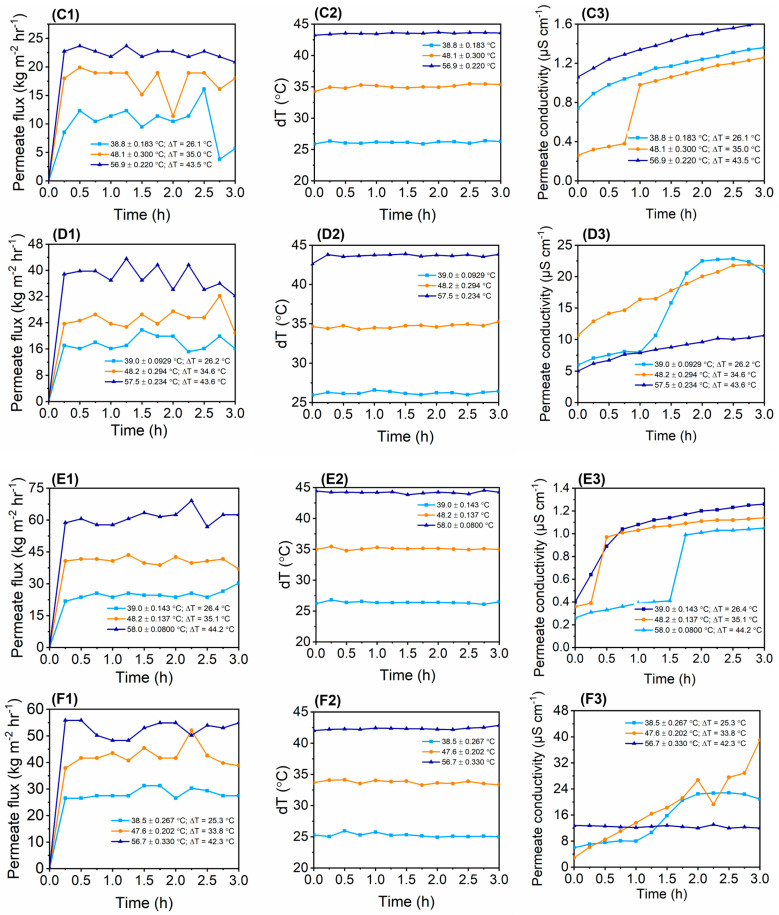
Permeate flux, deltaT, permeate conductivity vs. time at various temperatures for water desalination in DCMD: (**A1**–**A3**) M1, (**B1**–**B3**) M2, (**C1**–**C3**) M3, (**D1**–**D3**) M4, (**E1**–**E3**) PTFE-20, and (**F1**–**F3**) PTFE-45.

The 3.5 wt% NaCl was used to assess the MD process performance of the as-synthesised membranes (Figure 5). Evaluations were performed at three different feed temperatures to assess their effect on process performance. The permeate flux increased with an increase in feed temperature. At 40 ℃, the water fluxes of M1, M2, M3, M4, PTFE-20, and PTFE-45 were 7.58 kg m*^−^*^2^ h*^−^*^1^, 8.52 kg m*^−^*^2^ h*^−^*^1^, 11.36 kg m*^−^*^2^ h*^−^*^1^, 19.88 kg m*^−^*^2^ h*^−^*^1^, 25.68 kg m*^−^*^2^ h*^−^*^1^ and 27.46 kg m*^−^*^2^ h*^−^*^1^, respectively. Upon increasing the feed temperature to 60 ℃, the water fluxes increased to 14.20 kg m*^−^*^2^ h*^−^*^1^ (M1), 22.72 kg m*^−^*^2^ h*^−^*^1^ (M2), 22.69 kg m*^−^*^2^ h*^−^*^1^ (M3), 39.77 kg m*^−^*^2^ h*^−^*^1^ (M4), 62.5 kg m*^−^*^2^ h*^−^*^1^ (PTFE-20), and 54.92 kg m*^−^*^2^ h*^−^*^1^ (PTFE-45), respectively. The increase in feed temperature increased the vapour pressure gradient, thus improving the mass transfer (Figure 5A2–F2). These findings were substantiated by previously reported studies [31,61,62]. The permeate flux and deltaT remained stable for 3 hs, thus indicating process resistance to flux decays. Although the flux remained relatively stable, minimal fluctuations were recorded. For instance, the permeate flux of M4 was 38.83 kg m*^−^*^2^ h*^−^*^1^ at t = 15 min. Though deltaT remained relatively constant at t = 180 min, the flux slightly decreased to 32.20 kg m*^−^*^2^ h*^−^*^1^. These variations are caused by various parameters including pore wetting and concentration polarization [63]. Similar results were recorded for M3 where the permeate flux decreased from 22.72 kg m*^−^*^2^ h*^−^*^1^ to 20.83 kg m*^−^*^2^ h*^−^*^1^ at the same time intervals. The membrane conductivity increased slightly as a function of time for all operating temperatures. This increase in conductivity implied the slight transfer of water in the liquid state caused by membrane wetting. Interestingly, the rate of permeate conductivity increase was higher at high feed temperatures. This was associated with increased salt solubility. Although the membranes experienced slight wetting effects, the salt rejection remained relatively high (>99%) for all membranes. Therefore, the increased permeate conductivity effects were negligible as the membranes demonstrated the capacity to produce high-quality distillate. Comparatively, the salt rejections of the synthesized membranes were comparable to commercial membranes, thus motivating the successful incorporation of NPs into the membrane to improve process performance. Furthermore, the findings of this study were compared with the existing literature where PVDF membranes were modified with NPs for use in MD systems (Table 4). A comparative assessment elucidates the role of NPs towards process performance. Substantially, incorporation of NPs into the membrane matrix enhanced the membranes’ properties and their structural integrity. According to Ardeshiri et al. (2018), the incorporation of ZnONPs improved the porosity and surface roughness of PVDF membranes, thus ensuring high MD process performance [64]. Specifically, water flux (25 kg m*^−^*^2^ h*^−^*^1^) and salt rejection (99%) were reported. Other studies used modified SiO_2_NPs to improve membrane properties. Increased membrane porosity and WCA were reported [55,65]. These properties were instrumental in ensuring high permeate flux and salt rejection as seen in Table 4. In some instances, CNTs are used to modify PVDF membranes for DCMD, thus ensuring 100% salt rejection [39]. According to the existing findings, various operating conditions were evaluated to provide an overview of process versatility. These include process temperatures and flow rates. The water flux and salt rejection reported in the current study correspond to the existing literature at a feed temperature of 60 °C. Therefore, manipulation of the membrane properties for improved MD process performance is key.

## 4. Conclusions

This study explored membrane preparation and their modification through nanoparticle incorporation to improve the MD process performance. Synthetic membranes, namely, M1, M2, M3, and M4, were comparatively assessed against commercial membranes, PTFE-20 and PTFE-45. The membrane WCA decreased from 116.52° (M1) to 84.69° (M2) upon the incorporation of the pore former (PVP). However, the WCA increased significantly to 101.60° and 103.61° upon further addition of fCNTs (M3) and fSiO_2_NPs (M4), respectively. Furthermore, the LEP of the membranes decreased upon increased pore formation within the membrane surface. Interestingly, the incorporation of fCNTs and fSiO_2_NPs produced membranes of high LEP values > 500 kPa. Based on SEM analysis, these membranes were densely porous with irregular patterns of varying small, round, and spongy pores. After the addition of fCNTs in M3, the increased LEP was governed by reduced pore size of the membrane. In contrast, the incorporation of fSiO_2_NPs increased the size of the macrovoids and therefore indicated the dependence of M4 LEP on its hydrophobicity with a slight dependence on pore size. The as-synthesised membranes presented comparable properties to commercial membranes, thus demonstrating their potential applications in MD systems. However, further research is required to investigate the synergistic effect of the additives towards improved performance at the industrial level. This includes a recommended investigation into the fluorination of CNTs and their effect on process performance. During separation experiments, the as-synthesised membrane achieved >99.0% salt rejection and produced relatively stable fluxes and deltaT profiles. Based on flux, salt rejection tests, and physicochemical properties, the fSiO_2_NPs were the most favourable hydrophobic additive-producing membranes of high performance. The curation of membrane properties and structural integrity through the incorporation of fCNTs and fSiO_2_NPs provided a clear path towards improved wetting resistance. As a result, the current study provided experimental evidence for the successful use of modified PVDF membranes in DCMD, thus opening further research directions towards improved process performance.

## Figures and Tables

**Table 1:** Composition of the as-synthesised membranes.

Membrane	DMF	DMAc	PVDF (wt%)	PVP (wt%)	fCNTs (wt%)	fSiO_2_NPS (wt%)
M1	51.0	34.0	15.0	0.0	0.0	0.0
M2	50.9	34.0	15.0	0.1	0.0	0.0
M3	50.8	33.9	15.0	0.1	0.2	0.0
M4	48.7	32.5	15.0	0.1	0.2	3.5

**Table 2:** Physical characteristics of the as-synthesised PVDF and PTFE membranes.

Membrane	Porosity (%)	WCA (°)	LEP (kPa)	Pore Size (µm)
M1	81.68 ± 3.00	116.52	393 ± 4.00	0.27
M2	73.00 ± 5.14	84.69	273 ± 37.7	0.21
M3	64.34 ± 0.13	101.60	500 ± 97.9	0.19
M4	63.61 ± 0.95	103.81	590 ± 90.0	0.22
PTFE-20	54.13 ± 1.94	97.35	603 ± 20.5	0.20
PTFE-45	51.03 ± 1.48	101.57	200 ± 90.0	0.45

**Table 3:** Young’s modulus of the as-synthesised PVDF membranes and commercial PTFE membranes.

Membrane	Young’s Modulus
M1	0.45 ± 0.24
M2	1.06 ± 0.42
M3	1.07 ± 0.25
M4	0.39 ± 0.30
PTFE-20	2.90 ± 0.88
PTFE-45	6.96 ± 4.63

**Table 4:** Comparison of nanoparticle-modified PVDF membranes for water desalination in DCMD.

Membranes	Modifying NPs	Operating Conditions			Process Performance		Ref.
		Feed Temperature (°C)	Permeate Temperature (°C)	Flow Rate (mL min^−1^)	Flux (kg m^−2^ h^−1^)	Rejection (%)	
PVDF	ZnO	86	22	400.00	25.00	99	[64]
PVDF	Halloysite Nanotubes	60	20	252.36	5.52	95	[66]
PVDF nanofiber	SiO_2_NPs	20–80	20	750.00	34.2	99	[65]
PVDF	TiO_2_-SiO_2_	40	20	300.00	11.00	99	[55]
PVDF	CNTs	82	20	48.00	34.20	100	[39]
PVDF	fSiO_2_NPs/fCNTs	60	10	601.00	39.77	99	This study

## Data Availability

Data is available upon request.

## Supplementary Materials

The following supporting information can be downloaded at: https://www.mdpi.com/article/10.3390/membranes13030317/s1, Figure S1: FTIR spectra of unmodified and modified (A) SiO2NPs and (B) CNTs, Figure S2: FTIR spectra obtained for (A) all synthesised membranes (M1–M4) and (B) commercial membranes (PTFE-20 and PTFE-45), Figure S3: Pure water flux and deltaT vs time at various temperatures for (A1,A2) M1, (B1,B2) M2, (C1,C2) M3, (D1,D2) M4, (E1,E2) PTFE-20 and (F1,F2) PTFE-45. References [18,41,39,63,67,68,69,70,71] is cited in Supplementary Materials.
