# Supplementary material for: Nanoparticle-Enhanced PVDF Flat-Sheet Membranes for Seawater Desalination in Direct Contact Membrane Distillation
Source: Membranes (Basel). 2023 Mar 9;13(3):317. doi: 10.3390/membranes13030317 (PMC10052890; doi:10.3390/membranes13030317)
Supplement: Supplementary file 1 [file membranes-13-00317-s001.zip › membranes-2252499-supplementary.pdf]

membranes

Supplementary Materials

Nanoparticle-Enhanced PVDF Flat-Sheet Membranes for Seawater Desalination in Direct Contact Membrane Distillation

Indira Chimanlal [1,2], Lebea N. Nthunya [1,2], Oranso T. Mahlangu [3], Bastian Kirkebæk [2], Aamer Ali [2], Cejna A. Quist-Jensen [2,*] and Heidi Richards [1,*]

[1] Molecular Sciences Institute, School of Chemistry, University of Witwatersrand, Private Bag X3, Johannesburg 2050, South Africa; indira17.chimanlal@gmail.com (I.C.); nthunyalebea@gmail.com (L.N.N.)
[2] Center for Membrane Technology, Department of Chemistry and Bioscience, Aalborg University, Fredrik Bajers Vej 7H, 9220 Aalborg, Denmark; bski@bio.aau.dk (B.K.); aa@bio.aau.dk (A.A.)
[3] Institute for Nanotechnology and Water Sustainability, College of Science, Engineering and Technology, University of South Africa, Florida Science Campus, Johannesburg 1709, South Africa; orathem@gmail.com
* Correspondence: cejna@bio.aau.dk (C.A.Q.-J.); heidi.richards@wits.ac.za (H.R.)

1. Fourier Transform Infrared (FTIR) Analysis of the NPs and Membranes

1.1. The FTIR Analysis of the Nanoparticle Additives

The FTIR spectra of SiO_2NPs and fSiO_2NPs is presented in Figure S1A. A peak at 1454.23 cm^{-1} was associated to a Si-N stretch contributed by silica and nitrogen reaction from ammonium hydroxide. The appearance of a strong band at 1027.99 cm^{-1} and a medium band at 783.05 cm^{-1} was attributed to a Si-O-Si stretch and bend, respectively. A peak at 1602.74 cm^{-1} was assigned to an -OH bend caused by physical absorption of water [1]. In addition to this, a medium band at 933.48 cm^{-1} was associated to Si-OH bend. The absorption of water evidenced by a weak, broad peak at 3284.55 cm^{-1} was ascribed to an -OH stretch. Notably, the spectra for the SiO_2NPs and fSiO_2NPs did not display significant structural variation. This is because, the SiO_2NPs was physically modified with no chemical reaction. Thus, the coating process did not alter the chemical properties of the SiO_2NPs but rather changed particle size as evidenced by the TEM micrographs. Figure S1B presents the FTIR spectra of the CNTs. However, the spectra did not show significant differences to fCNTs. Notably, a peak at 1582.04 cm^{-1} and 1487.08 cm^{-1} represented an O-H bend due to chemically absorbed water [2], and a CH_2 bend respectively. Gao et al. (2020) reported some differences in NP spectra caused by flourination. In their spectra, the appearance of the peaks at 1080 and 1270 cm^{-1} were referred to a Si-O-Si and C-F bands, respectively [2]. Minor differences were caused by the small addition of silane reagents leading to insignificant fluorination. Though a small addition of silane reagents reduces production cost of modified CNTs, required properties such as hydrophobicity were compromised. Therefore, optimization processes are required to establish a trade-off between production costs and improved process performance during application.

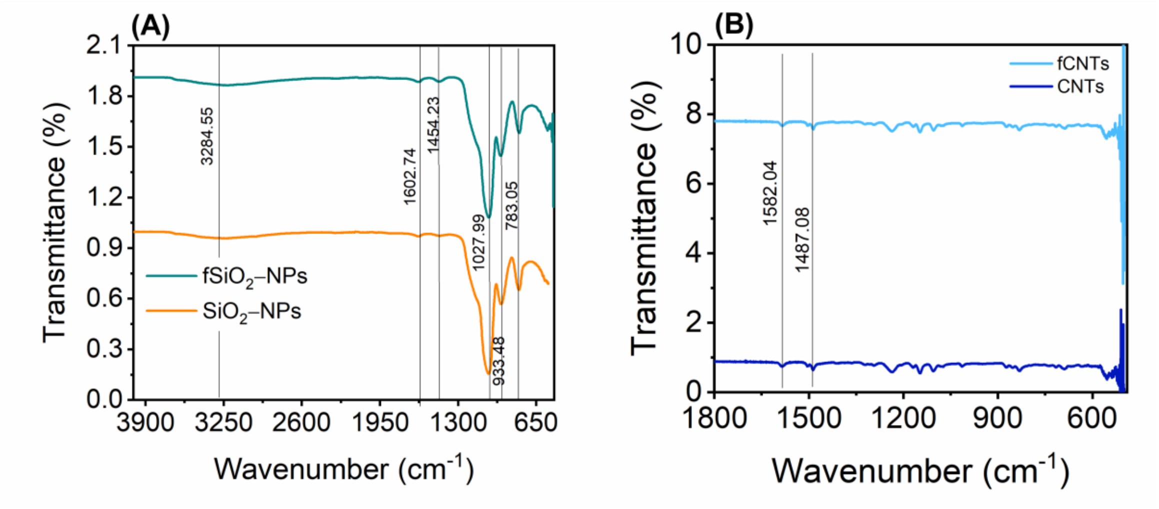

Figure S1. FTIR spectra of unmodified and modified (**A**) SiO_2NPs and (**B**) CNTs.

1.2. The FTIR Analysis of the As-Synthesized Membranes

FTIR of the as-synthesised and commercial membranes is presented in Figures S2A,B. The as-synthesised membranes share similar peaks corresponding to bonds characteristic of the PVDF base polymer (Figure S2A). The bands at 1398.48 cm^{-1} and 1169.21 cm^{-1} are assigned to C-F stretch, and C-C vibrations. Similar findings were reported previously [3,4]. Additionally, bands at 1069.93–836.37 cm^{-1} were associated to scissoring vibration of H_2C-CF_2 and CH_2 rocking vibrations, respectively [4]. Also, the FTIR spectra for M2–M4 did not vary significantly from that of M1 despite the inclusion of membrane additives due to physical interactions rather than chemical modifications. Upon analyzing PTFE-20, two distinct peaks at 1205.63 and 1149.21 cm^{-1} corresponding to asymmetric and symmetric CF_2 stretching, respectively were observed. In the case of PTFE-45, similar peaks were recorded at 1198.49 and 1146.35 cm^{-1} respectively. Furthermore, peaks appearing range of 640–552 cm^{-1} were ascribed to CF_2 wagging and bending vibrations [5].

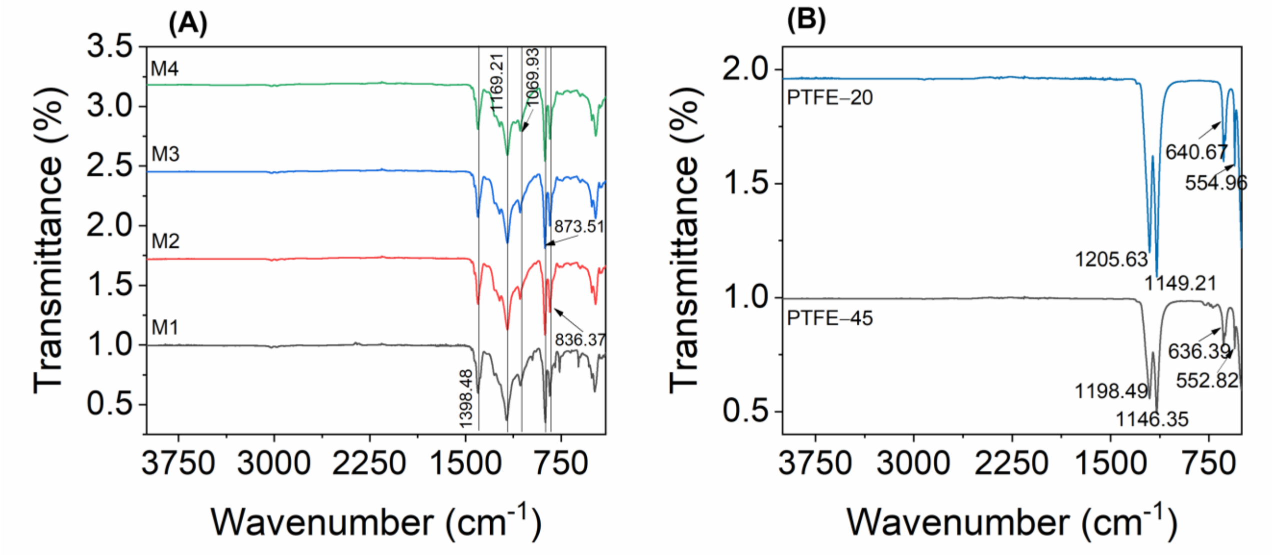

Figure S2. FTIR spectra obtained for (**A**) all synthesised membranes (M1–M4) and (**B**) commercial membranes (PTFE-20 and PTFE-45).

1.3. Pure Water Flux Determination in DCMD

Pure water filtration tests were carried out to evaluate MD process performance in the absesnce of dissolved solutes (Figure S3). Evaluations were performed at three different feed temperatures to assess their effect on process performance. The permeate flux (at feed temperature of 40 °C) of M1, M2, M3, M4, PTFE-20 and PTFE-45 were 1.89 kg m^{-2} h^{-1}, 1.26 kg m^{-2} h^{-1}, 0.63 kg m^{-2} h^{-1}, 2.21 kg m^{-2} h^{-1}, 4.42 kg m^{-2} h^{-1}, and 6.94 kg m^{-2} h^{-1} respectively. Upon increase the feed temperature to 60 °C, the permeate flux increased to 4.10 kg m^{-2} h^{-1}, 4.73 kg m^{-2} h^{-1}, 1.89 kg m^{-2} h^{-1}, 5.05 kg m^{-2} h^{-1}, 6.62 kg m^{-2} h^{-1}, and 13.88 kg m^{-2} h^{-1} respectively. The increase in feed temperature increased vapour pressure gradient resulting high process driving force [6,7]. Similar findings were reported by Edwie and Chung (2013) at feed temperature range of 40–70 °C. Notably, increased feed temperature does not only improves rate of water recovery, but also the process thermal efficiency [8]. DeltaT increased significantly upon an increase in feed temperature (Figure S3A2–F2), thus translating to the increase in permeate flux [7]. PTFE-45 presented higher flux compared to PTFE-20, thus indicating the role played by the membrane pore size. Also, membrane modifications in order of M1 to M4 presented a progressive increase in permeate flux with some discrepancies for M3. The increased flux at 60 °C from M1 (4.10 kg m^{-2} h^{-1}) to M2 (4.73 kg m^{-2} h^{-1}) was associated to large pore size caused by incorporation of PVP (a pore former). However, incorporation of fCNTs (M3) caused a decline in water flux (1.89 kg m^{-2} h^{-1}) due pore blockage. The M4 presented the highest permeate flux (5.05 kg m^{-2} h^{-1}) compared to other synthetic membranes. Incorporation of fCNTs and fSiO_2NPs improved membrane hydrophobicity significantly, thus minimizing mass transfer resistance by ensuring exclusive vapour transport without competing water molecules in liquid state [9]. Moreover, the permeate flux obtained for M4 was relatively comparable PTFE-20, suggesting its promising performances similarly to a commercial membrane.

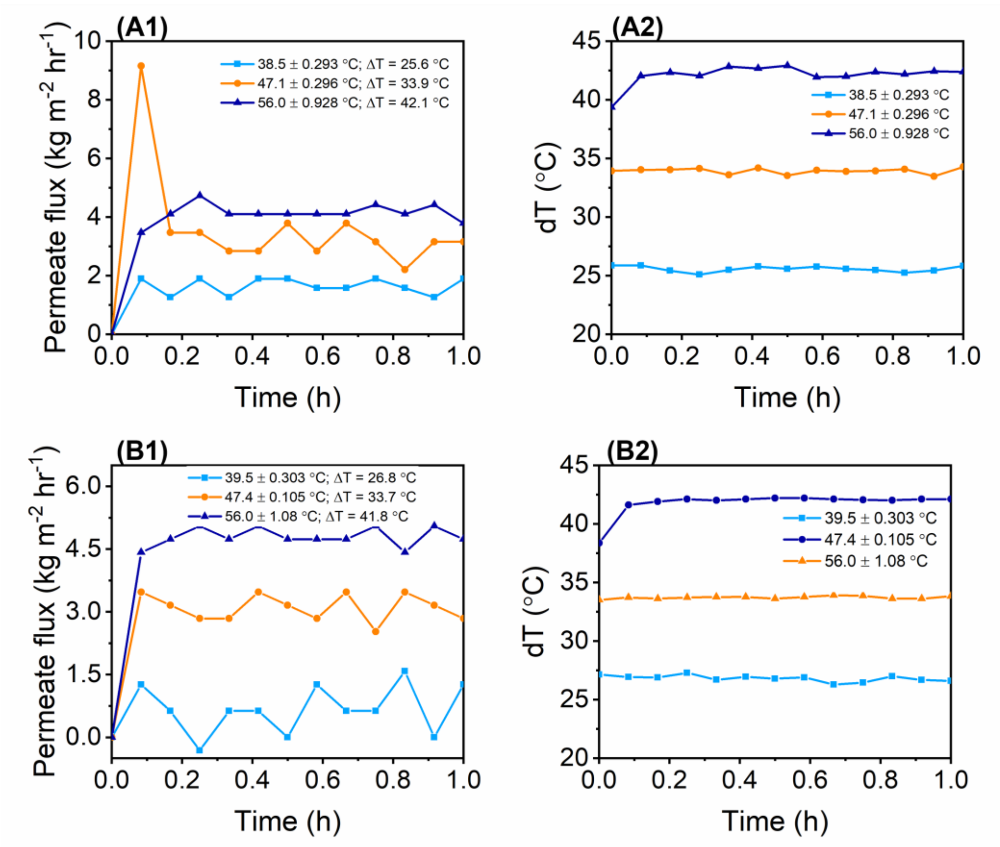

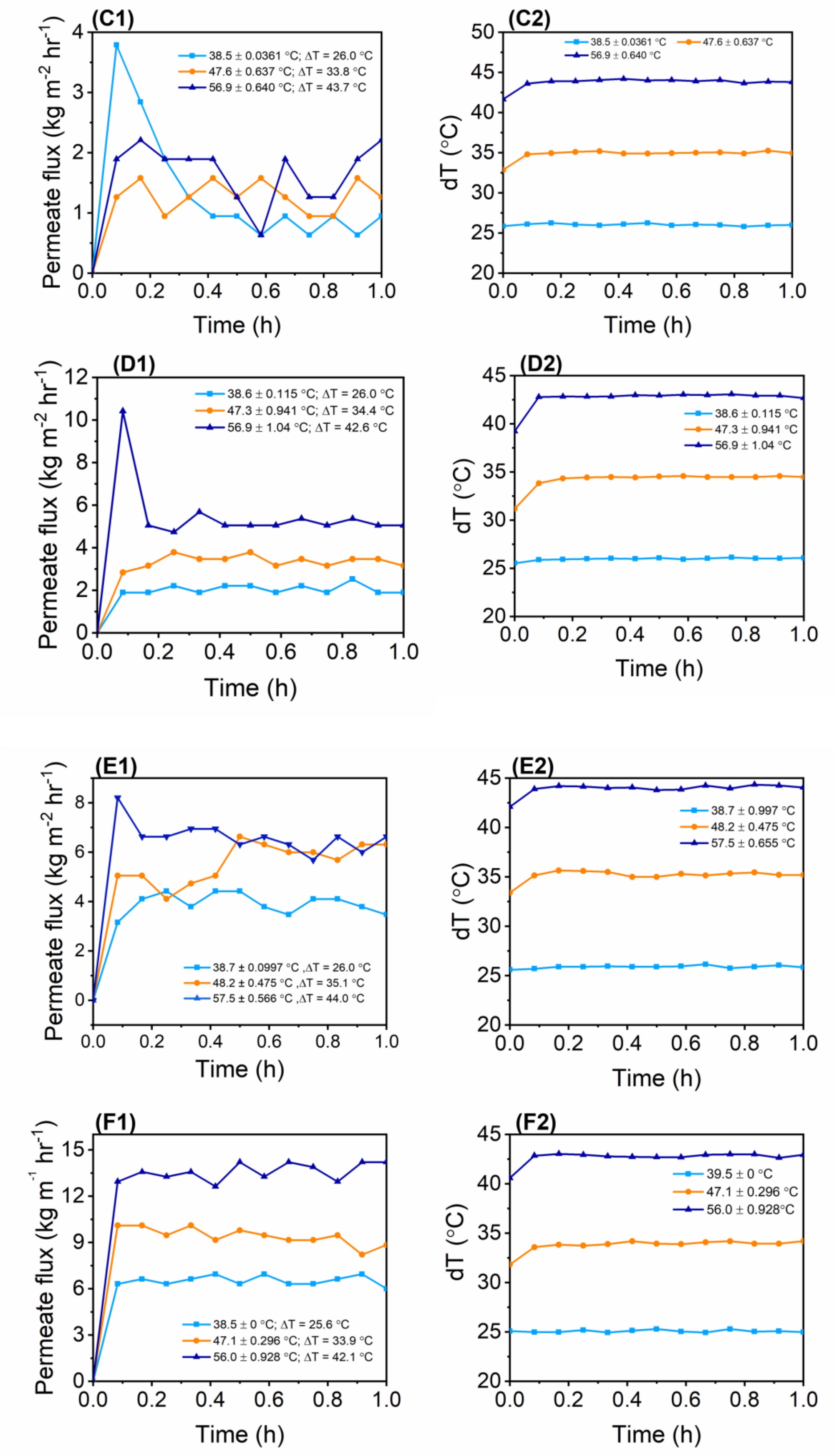

Figure S3. Pure water flux and deltaT vs time at various temperatures for (**A1,A2**) M1, (**B1,B2**) M2, (**C1,C2**) M3, (**D1,D2**) M4, (**E1,E2**) PTFE-20 and (**F1,F2**) PTFE-45.

References

1. Cai Y, Li J, Yi L, Yan X, Li J. Applied Surface Science Fabricating superhydrophobic and oleophobic surface with silica nanoparticles modified by silanes and environment-friendly fluorinated chemicals. *Appl Surf Sci.* **2018**, *450*, 102–11.
2. Gao, C.; Deng, W.; Pan, F.; Feng, X.; Li, Y. Superhydrophobic Electrospun PVDF Membranes with Silanization and Fluorosilanization Co-functionalized CNTs for Improved Direct Contact Membrane Distillation. *Eng. Sci.* **2020**, *9*, 35–43.
3. Silva, T.L.; Morales-Torres, S.; Figueiredo, J.L.; Silva, A.M. Multi-walled carbon nanotube/PVDF blended membranes with sponge- and finger-like pores for direct contact membrane distillation. *Desalination* **2015**, *357*, 233–245
4. Puspitasari, V.; Granville, A.; Le-Clech, P.; Chen, V. Cleaning and ageing effect of sodium hypochlorite on polyvinylidene fluoride (PVDF) membrane. *Sep. Purif. Technol.* **2010**, *72*, 301–308
5. Wang, H.; Wen, Y.; Peng, H.; Zheng, C.; Li, Y.; Wang, S.; Sun, S.; Xie, X.; Zhou, X. Grafting Polytetrafluoroethylene Micropowder via in Situ Electron Beam Irradiation-Induced Polymerization. *Polymers* **2018**, *10*, 503.
6. Gryta, M. Separation of saline oily wastewater by membrane distillation. *Chem. Pap.* **2020**, *74*, 2277–2286
7. Quist-Jensen, C.A.; Macedonio, F.; Horbez, D.; Drioli, E. Reclamation of sodium sulfate from industrial wastewater by using membrane distillation and membrane crystallization. *Desalination* **2017**, *401*, 112–119
8. Edwie, F.; Chung, T.-S. Development of simultaneous membrane distillation–crystallization (SMDC) technology for treatment of saturated brine. *Chem. Eng. Sci.* **2013**, *98*, 160–172
9. Dumée, L.F.; Gray, S.; Duke, M.; Sears, K.; Schutz, J.; Finn, N. The role of membrane surface energy on direct contact membrane distillation performance. *Desalination* **2013**, *323*, 22–30
